# Surgical stabilization of rib fractures in older adults: a retrospective cohort study

**DOI:** 10.1007/s13304-026-02597-8

**Published:** 2026-03-19

**Authors:** Erhan Özer, Teoman Ekim

**Affiliations:** 1 Department of Thoracic Surgery, Bursa Uludağ University Faculty of Medicine Hospital, Bursa, Turkey; 2 Department of Thoracic Surgery, Bursa Çekirge State Hospital, Bursa, Turkey

**Keywords:** Rib fractures, Geriatric trauma, Surgical stabilization, Chronic pain

## Abstract

The role of surgical stabilization of rib fractures (SSRF) in older adults remains uncertain, particularly regarding functional recovery and long-term pain. This study examined whether chronological age influences postoperative outcomes and evaluated the performance of injury-severity scoring systems in geriatric patients undergoing SSRF. This retrospective cohort included 524 adults who underwent SSRF between 2012 and 2023. Patients were stratified into non-geriatric (< 65 years, *n* = 331) and geriatric (≥ 65 years, *n* = 193) groups. Demographics, injury characteristics, perioperative outcomes, pain trajectories, and functional recovery were compared. Predictors of postoperative complications and chronic pain were assessed using univariate and multivariate logistic regression. Correlations between clinical outcomes and RibScore, AIS-Thorax, and BPC18 were analyzed using Spearman coefficients. Geriatric patients were more often injured by low-energy falls and had higher rates of pulmonary contusion (74.6% vs. 63.9%) yet demonstrated complication rates similar to younger adults (14.0% vs. 12.7%, *p* = 0.696). Pain trajectories were comparable across all follow-up intervals. Functional recovery was slower in older adults, who required an additional month to return to baseline activity (median 3 vs. 2 months, *p* < 0.001). The number of fractured ribs on the operated side independently predicted postoperative complications (OR 1.863, 95% CI 1.396–2.487, *p* < 0.001), whereas age was not an independent predictor. Longer time-to-surgery (OR 1.341, *p* = 0.001), longer hospitalization (OR 1.059, *p* = 0.044), and postoperative complications (OR 1.941, *p* = 0.036) independently predicted chronic pain. AIS-Thorax showed the strongest correlations with clinical outcomes, while all three scoring systems demonstrated weak associations with postoperative pain. In appropriately selected patients, SSRF provides comparable safety and pain recovery across age groups. Although functional recovery is slower in older adults, overall outcomes remain favorable. These findings support shifting from age-based to physiology-based decision-making for SSRF.

**Supplementary Information:**

The online version contains supplementary material available at 10.1007/s13304-026-02597-8.

## Introduction

Rib fractures are among the most common injuries following blunt thoracic trauma, and their clinical impact becomes more pronounced with advancing age [1, 2]. Older adults not only sustain chest wall injuries more frequently, but also face diminished physiological reserve, multimorbidity, and an increased susceptibility to pulmonary complications [3, 4]. Recent epidemiological trends further highlight the growing role of low-energy mechanisms—particularly ground-level falls—as a dominant cause of rib fractures in the geriatric population, marking a shift from the high-energy trauma patterns previously described.

Surgical stabilization of rib fractures (SSRF) has gained widespread acceptance for selected indications, supported by recent guidelines from the Chest Wall Injury Society and the World Society of Emergency Surgery [5–7]. These recommendations outline clear indications—particularly for flail chest and markedly displaced fractures—while acknowledging areas where evidence remains limited. Among these uncertainties, the role, safety, and functional benefit of SSRF in geriatric patients remain poorly defined. Despite increasing adoption of SSRF, older adults remain underrepresented in prospective trials [8, 9]. Existing studies provide conflicting results due to small sample sizes, heterogeneous injury patterns and frailty profiles, and inconsistent surgical selection criteria. Consequently, whether chronological age should influence surgical decision-making remains uncertain, and clinicians often hesitate to offer SSRF to older adults due to concerns regarding higher complication rates, slower recovery, or prolonged pain—perceptions not consistently supported by current data [10, 11].

The present study addresses these gaps by comprehensively comparing postoperative complications, pain trajectories, and functional recovery between geriatric and non-geriatric patients in a large, contemporary cohort. We additionally evaluate the comparative performance of three widely used injury-severity scoring systems and identify predictors of chronic pain in older adults. We hypothesize that physiological reserve and injury burden—rather than chronological age—are the primary determinants of postoperative risk.

## Patients and methods

### Study design and patient selection

This retrospective cohort study included patients who had SSRF at a single tertiary referral center between January 2012 and December 2023. Out of 737 patients, 213 (28.9%) were excluded: severe traumatic brain injury (GCS ≤ 8, *n* = 89), hemodynamic instability requiring emergency thoracotomy (*n* = 31), death within 24 h (*n* = 18), insufficient follow-up data (*n* = 52), and incomplete records (*n* = 23). The final group comprised 524 patients, divided into geriatric (≥ 65 years, *n* = 193) and non-geriatric (< 65 years, *n* = 331) groups. Only surgically treated patients were included, and no non-operative control group was available; therefore, comparative effectiveness between SSRF and conservative management cannot be evaluated.

All patients presenting with traumatic rib fractures were evaluated by a multidisciplinary trauma team, and the decision to perform surgical stabilization was based on standardized institutional criteria comprising primary and secondary indications. Primary indications included flail chest involving more than two rib segments with respiratory compromise, non-flail fractures with marked bicortical displacement of three or more adjacent ribs, and progressive chest wall deformity resulting in impaired respiratory mechanics. Secondary indications consisted of respiratory failure requiring intubation primarily due to chest wall injury, persistent pain impairing ventilation despite 48–72 h of optimal conservative therapy, clinically evident chest wall instability on examination, and inability to participate in physiotherapy because of uncontrolled pain or mechanical instability.

Upon admission, each patient underwent chest computed tomography within 24 h to evaluate fracture configuration, displacement, and associated thoracic injuries. Standard trauma management was applied, including multimodal analgesia, pulmonary physiotherapy, and close respiratory monitoring. Endotracheal intubation and mechanical ventilation were initiated only when clinically indicated for respiratory insufficiency or concomitant injuries; no patient was intubated solely to facilitate surgical fixation.

### Surgical technique, material selection, and perioperative management

Surgical stabilization was performed under general anesthesia by an experienced thoracic surgery team. Patients who required concurrent thoracoscopic intervention—such as for hemothorax or pneumothorax—were intubated with a double-lumen endotracheal tube, while others underwent single-lumen intubation. The surgical position was individualized according to the fracture location and the presence of associated injuries, such as spinal or pelvic trauma.

All fixations were performed using the IAWA Nitinol Fixation System (Yanzhou Industrial Park, Jiangsu, China), which employs thermally activated memory metal plates and clips that adapt to the natural rib contour, eliminating the need for screw fixation. A muscle-sparing incision was preferred whenever possible, and the fracture lines were exposed with meticulous dissection of the intercostal muscles to preserve the neurovascular bundle and prevent nerve entrapment along the clip line. After anatomical reduction, nitinol plates were applied over the rib surface and secured with at least two clips per plate.

When indicated, video-assisted thoracoscopy was used for evacuation of hemothorax or pneumothorax and inspection of the pleural cavity. Wounds were closed in layers, and a closed-suction drain was inserted into the subcutaneous tissue. If pleural violation occurred, a chest tube was placed intra-thoracically. Postoperatively, patients were transferred either to the intensive care unit or to the ward based on clinical assessment, as no routine ICU admission protocol was applied.

Pain control followed a standardized multimodal analgesia protocol consisting of intravenous opioids (morphine or tramadol) and non-steroidal anti-inflammatory drugs (NSAIDs). In selected cases, regional anesthesia techniques such as thoracic epidural or paravertebral blocks were used upon anesthesiology consultation. Early postoperative rehabilitation included incentive spirometry, deep-breathing exercises, and mobilization under supervision of the physiotherapy team to promote optimal pulmonary recovery. Supplemental oxygen was administered when required. Non-invasive ventilatory support (high-flow nasal oxygen or non-invasive ventilation) was used in patients with transient respiratory insufficiency. Endotracheal intubation and mechanical ventilation were reserved for patients who developed respiratory failure, severe pulmonary complications, or required airway protection due to associated injuries. No patient was intubated solely to facilitate surgical fixation.

### Data collection and outcome measures

Demographic, clinical, and injury-related data were retrospectively obtained from institutional electronic medical records. For each patient, trauma severity indices—including the Abbreviated Injury Scale for the thorax (AIS-Thorax), RibScore, and Blunt Pulmonary Contusion Score (BPC18)—were calculated based on admission chest computed tomography (CT) scans. Surgical parameters such as time from injury to operation, number of plates and clips used, duration of drainage, and total hospital length of stay were also recorded. Follow-up assessments were conducted at 1, 3, and 6 months after surgery. Pain intensity was measured on a 0–10 numerical rating scale, and functional recovery was evaluated through in-person or structured telephone interviews performed by the surgical team.

The primary outcome was total hospital length of stay.

Secondary outcomes included postoperative complications, intensive care unit (ICU) stay, postoperative pain scores at 1, 3, and 6 months, the incidence of chronic pain (defined as pain persisting beyond six months), time to return to pre-injury functional status, and in-hospital mortality. Comparisons were made between geriatric (≥ 65 years) and non-geriatric (< 65 years) patient groups to identify the effect of age on clinical and functional outcomes.

### Variables

Pulmonary contusions were quantified using the Blunt Pulmonary Contusion Score (BPC18), calculated from axial and coronal CT images with a slice thickness ≤ 3.5 mm. Each lung was divided into three zones (upper, middle, and lower), and the degree of parenchymal opacification was graded visually as ≤ 33%, 33–66%, or > 66%, corresponding to scores of 1, 2, and 3, respectively. The total BPC18 score, summing all six zones, ranged from 0 to 18, with a maximum of 9 points per lung. [12].

Rib fracture severity was assessed using the RibScore, a composite index assigning one point for each of the following features: ≥6 rib fractures, bilateral fractures, flail chest, ≥ 3 bicortically displaced fractures, first rib fracture, and fractures involving all three anatomic regions (anterior, lateral, posterior) [13].

The AIS-Thorax was also used to classify injury severity from minor to critical, as follows: score 1, single rib fracture or sternal contusion; score 2, 2–3 rib fractures or sternal fracture; score 3, ≥ 4 displaced fractures or open fracture; score 4, flail chest without respiratory failure; and score 5, flail chest requiring ventilatory support [14].

### Functional outcome definition

Functional recovery was defined as the ability to return to pre-injury activity level, adjusted for age and lifestyle. In younger adults, this referred to resuming occupational and recreational activities, while in older adults, it denoted the ability to return to their prior living arrangements and independently perform daily activities. Recovery was considered satisfactory when pain was not a limiting factor for routine physical activity, corresponding to a Visual Analog Scale (VAS) score of 3 or less.

### Statistical analysis

Continuous variables were tested for normality using the Shapiro–Wilk method and are presented as mean ± standard deviation or median (interquartile range), as appropriate. Group comparisons between geriatric (≥ 65 years) and non-geriatric (< 65 years) patients were performed using the independent samples t-test or Mann–Whitney U test for continuous variables, and the χ² test or Fisher’s exact test for categorical variables.

Correlation analyses between injury severity indices (AIS-Thorax, RibScore, and BPC18) and clinical outcomes—including ICU stay, total hospital stay, postoperative pain scores, and functional recovery time—were performed using Spearman’s rank correlation coefficients.

To identify predictors of adverse outcomes, univariate logistic regression analyses were conducted separately for postoperative complications and chronic pain (persistent pain > 6 months). Variables demonstrating statistical significance in univariate analysis were subsequently entered into multivariate logistic regression models to determine independent predictors. Odds ratios (OR) and 95% confidence intervals (CI) were calculated for all regression analyses.

A two-tailed p-value < 0.05 was considered statistically significant. All statistical analyses were performed using SPSS version 29 (IBM Corp., Armonk, NY, USA).

### Ethical considerations

This study was approved by the Institutional Review Board (IRB) of Uludag University (Approval No: [2024-16/24]). Written informed consent was obtained from all patients or their legal representatives. The study was conducted in accordance with the principles of the Declaration of Helsinki.

## Results

Among the 524 patients, 331 (63.2%) were younger than 65 years and 193 (36.8%) were aged ≥ 65 years. The population was predominantly male (82.6%), with a mean age of 59.5 years (range: 23–95). Baseline characteristics and injury severity scores were comparable across age groups (Table 1).

**Table 1:** Demographic Characteristics, Injury Mechanisms, and Injury Severity Profiles of the Study Cohort

Characteristic	Non-Geriatric (< 65 years) *n* = 331	Geriatric (≥ 65 years) *n* = 193	*p*-value
Age, mean (SD)	51.62 (11.2)	72.8 (6.8)	< 0.001
Male sex, n (%)	275 (83.1)	158 (81.9)	0.736
BMI, mean (SD)	26.29 (4.1)	26.17 (3.9)	0.732
Injury Mechanism, n (%)			
Fall	153 (46.2)	126 (65.3)	< 0.001
Traffic accident	124 (37.5)	52 (26.9)	0.016
Injury Characteristics			
Number of ribs fractured, median (IQR)	5 (1–10)	5 (1–10)	0.847
Flail chest, n (%)	56 (16.9)	26 (13.5)	0.295
Bilateral fractures, n (%)	57 (17.2)	31 (16.1)	0.746
First rib fracture, n (%)	45 (13.6)	17 (8.8)	0.112
Intrathoracic Complications			
Pulmonary contusion, n (%)	212 (64.0)	144 (74.6)	0.017
Pneumothorax, n (%)	100 (30.2)	52 (26.9)	0.436
Hemothorax, n (%)	183 (55.3)	115 (59.6)	0.348
Injury Severity Scores			
AIS-Thorax, mean (SD)	2.93 (0.8)	3.0 (0.7)	0.604
RibScore, mean (SD)	1.94 (1.1)	1.93 (1.0)	0.912
BPC18, mean (SD)	1.37 (1.8)	1.46 (1.9)	0.351

Abbreviations: BMI, Body Mass Index; AIS-Thorax, Abbreviated Injury Scale—Thorax; BPC18, Blunt Pulmonary Contusion Score; SD, Standard deviation; IQR, Interquartile range

**Table 2:** Comparison of operative characteristics and postoperative outcomes between geriatric and non-geriatric patients

Outcome	Non-Geriatric *n* = 331	Geriatric *n* = 193	*p*-value
Operative Characteristics			
Time to surgery, median days (IQR)	2 (1–10)	2 (1–7)	0.845
Number of ribs operated, mean (SD)	4.13 (2.1)	4.5 (2.3)	0.016
Number of plates used, median (IQR)	4.21 (2.8)	4.65 (3.1)	0.022
Bilateral surgery, n (%)	8 (2.4)	4 (2.1)	1.000
Postoperative Outcomes			
Overall complications, n (%)	42 (12.7)	27 (14.0)	0.696
Pneumonia	14 (4.2)	13 (6.7)	0.211
Hardware complications	16 (4.8)	6 (3.1)	0.342
Surgical site infection	3 (0.9)	5 (2.6)	0.151
ICU admission, n (%)	91 (27.5)	51 (26.4)	0.775
ICU stay, mean days (SD)	1.5 (3.2)	1.41 (2.8)	0.634
Hospital stay, mean days (SD)	8.68 (6.4)	8.79 (5.9)	0.703
30-day readmission, n (%)	16 (4.8)	8 (4.1)	0.711
In-hospital mortality, n(%)	9 (2.7)	9 (4.7)	0.239

**Table 3:** Comparison of postoperative pain scores and functional recovery between geriatric and non-geriatric patients

Outcome	Non-Geriatric *n* = 331	Geriatric *n* = 193	*p*-value
Pain Scores (0–10 scale)			
1-month, mean (median)	2.6 (3)	2.63 (3)	0.595
3-month, mean (median)	1.35 (1)	1.4 (1)	0.524
6-month, mean (median)	0.61 (0)	0.61 (0)	0.780
Functional Outcomes			
Chronic pain (> 6 months), n (%)	56 (16.9)	38 (19.7)	0.438
Return to activity, median months	2	3	< 0.001

**Table 4:** Correlation Analysis Between Injury Severity Indices (BPC18, RibScore, AIS-Thorax) and Key Clinical Outcomes

Clinical Outcome	BPC18 *r* / *p*-value	RibScore *r* / *p*-value	AIS-Thorax *r* / *p*-value
Hospital Outcomes			
ICU length of stay	0.363 / <0.001	0.318 / <0.001	0.340 / <0.001
Total hospital stay	0.316 / <0.001	0.283 / <0.001	0.248 / <0.001
Return to normal activity	0.114 / 0.813	0.175 / 0.017	0.204 / 0.001
Pain Outcomes			
1st month pain score	0.073 / 0.194	0.125 / 0.026	0.105 / 0.060
3rd month pain score	0.051 / 0.359	0.177 / 0.010	0.147 / 0.080
6th month pain score	0.032 / 0.571	0.194 / 0.008	0.134 / 0.074
Binary Outcomes (p-value)			
Chronic pain presence	0.382	0.027	0.025
Postoperative complications	0.045	0.027	0.346
In-hospital mortality	0.252	0.220	0.222

Abbreviations: r = Spearman correlation coefficient; BPC18 = Blunt Pulmonary Contusion Score; AIS = Abbreviated Injury Scale; ICU = intensive care unit. Bold values indicate statistical significance (*p* < 0.05)

Several variables showed univariate associations with chronic pain: delayed surgery (OR 1.425, 95% CI 1.202–1.690, *p* < 0.001), number of fractured ribs on the operated side (OR 1.154, 95% CI 1.020–1.307, *p* = 0.023), total hospital length of stay (OR 1.091, 95% CI 1.038–1.146, *p* < 0.001), postoperative complications (OR 2.316, 95% CI 1.304–4.114, *p* = 0.004), and AIS-Thorax score (OR 1.418, 95% CI 1.004–2.003, *p* = 0.047).

In multivariate analysis, only three variables retained significance: day of surgery after admission (OR 1.341, 95% CI 1.123–1.602, *p* = 0.001), total hospital stay (OR 1.059, 95% CI 1.002–1.119, *p* = 0.044), and postoperative complications (OR 1.941, 95% CI 1.043–3.610, *p* = 0.036). Neither the number of fractured ribs (OR 1.025, *p* = 0.809) nor AIS-Thorax (OR 1.160, *p* = 0.599) remained independent predictors.

Multiple clinical and radiologic parameters were associated with postoperative complications in univariate analyses, including the number of fractured ribs on the operated side (OR 1.400, 95% CI 1.219–1.610, *p* < 0.001), preoperative ventilator use (OR 1.543, 95% CI 1.023–2.328, *p* = 0.039), total number of plates (OR 1.321, 95% CI 1.120–1.558, *p* = 0.001), RibScore (OR 1.232, 95% CI 1.058–1.434, *p* = 0.007), BPC18 (OR 1.353, 95% CI 1.084–1.688, *p* = 0.007), first rib fracture (OR 3.119, 95% CI 1.599–6.086, *p* < 0.001), and male sex (OR 1.874, 95% CI 1.032–3.402, *p* = 0.039).

When these variables were entered simultaneously into the multivariate model, only the number of fractured ribs on the operated side (OR 1.863, 95% CI 1.396–2.487, *p* < 0.001) remained an independent predictor of complications. Preoperative ventilator use, plating burden, injury severity scores (RibScore and BPC18), first rib fracture, and sex lost statistical significance, indicating that complication risk is primarily driven by fracture burden rather than demographic or radiologic severity metric.

**Table 5:** Predictors of Chronic Pain and Postoperative Complications: Univariate and Multivariate Logistic Regression Analysis

Variable	Univariate OR (95% CI)	*p*-value	Multivariate OR (95% CI)	*p*-value
Chronic Pain Predictors				
Day of surgery after admission	1.425 (1.202–1.690)	< 0.001	1.341 (1.123–1.602)	0.001
No. of fractured ribs (operated side)	1.154 (1.020–1.307)	0.023	1.025 (0.839–1.252)	0.809
Total hospital length of stay	1.091 (1.038–1.146)	< 0.001	1.059 (1.002–1.119)	0.044
Postoperative complications	2.316 (1.304–4.114)	0.004	1.941 (1.043–3.610)	0.036
AIS-Thorax	1.418 (1.004–2.003)	0.047	1.160 (0.666–2.021)	0.599
Complication Predictors				
No. of fractured ribs (operated side)	1.400 (1.219–1.610)	< 0.001	1.863 (1.396–2.487)	< 0.001
Preoperative ventilator use	1.543 (1.023–2.328)	0.039	NS	NS
Total number of plates	1.321 (1.120–1.558)	0.001	NS	NS
RibScore	1.232 (1.058–1.434)	0.007	NS	NS
BPC18	1.353 (1.084–1.688)	0.007	NS	NS
First rib fracture	3.119 (1.599–6.086)	< 0.001	NS	NS
Male sex	1.874 (1.032–3.402)	0.039	NS	NS

Abbreviations: OR = Odds Ratio; CI = Confidence Interval; AIS = Abbreviated Injury Scale; BPC18 = Blunt Pulmonary Contusion Score; NS = Not significant in multivariate model

## Discussion

This study represents one of the largest comparative evaluations of SSRF in elderly patients, including 193 geriatric (≥ 65 years) and 331 younger individuals. Our findings challenge the traditional assumption that advanced age functions as an independent surgical risk factor. Despite higher pulmonary contusion rates (74.6% vs. 63.9%) and a greater number of stabilized ribs (4.5 vs. 4.1), geriatric patients experienced complication rates, ICU utilization, pain trajectories, and mortality comparable to younger adults. Importantly, this study provides the first comprehensive characterization of age-stratified pain trajectories, quantifies delays in functional recovery, compares the prognostic performance of three injury-severity scoring systems, and identifies modifiable predictors of chronic pain. Collectively, these findings support transitioning from age-based exclusion to patient selection guided by physiological reserve and injury burden. **This cohort represents surgically selected patients; frail elderly individuals with severely limited physiological reserve or early trauma-related mortality were not candidates for SSRF and are therefore not represented. Accordingly**,** these findings may not be generalizable to the entire geriatric trauma population**.

Injury patterns in our cohort reflect a notable shift toward low-energy trauma as the predominant mechanism in elderly patients, with falls accounting for nearly two-thirds of geriatric cases. This contrasts with earlier studies where high-energy motor vehicle collisions were the primary etiology in older adults(1). Pulmonary contusions occurred significantly more frequently in the geriatric group (74.6% vs. 63.9%), consistent with prior observations of heightened parenchymal vulnerability in aged lungs [15]. Despite this increased physiological burden, postoperative outcomes remained comparable between age groups, suggesting that timely surgical stabilization may effectively mitigate the respiratory complications traditionally attributed to advanced age.

Among the three injury severity indices evaluated, AIS-Thorax demonstrated the strongest correlations with clinical outcomes, particularly ICU length of stay (*r* = 0.340, *p* < 0.001) and total hospitalization (*r* = 0.248, *p* < 0.001). In contrast, RibScore and BPC18 showed only modest and inconsistent correlations across parameters, suggesting limited discriminatory value in this surgically managed cohort. Notably, all three indices exhibited weak correlations with postoperative pain (*r* < 0.20), indicating that pain intensity after SSRF does not directly reflect radiographic injury severity. From a clinical perspective, these findings suggest that AIS-Thorax may assist in early risk stratification and resource planning, whereas injury severity scores alone appear insufficient to guide surgical indication, timing of intervention, or prediction of long-term pain outcomes. Therefore, surgical decision-making should continue to rely primarily on clinical assessment of respiratory compromise, chest wall instability, and overall physiological reserve.

The observed complication rate of 14.0% in geriatric patients aligns with contemporary SSRF literature and compares favorably with historical conservative management outcomes [10, 11]. Although elderly patients underwent fixation of a slightly greater number of ribs (4.5 vs. 4.1), complication rates remained comparable between age groups (14.0% vs. 12.7%, *p* = 0.696). Multivariate analysis revealed that the number of fractured ribs on the operated side was the sole independent predictor of postoperative complications (OR 1.863, 95% CI 1.396–2.487, *p* < 0.001), while age, plating burden, injury severity scores (RibScore, BPC18), first rib fracture, and sex lost statistical significance. This finding reinforces that complication risk is driven primarily by fracture burden rather than chronological age or demographic factors, supporting physiologic rather than age-based patient selection for SSRF.

Functional recovery followed a trajectory distinct from pain outcomes; elderly patients required approximately one additional month to return to baseline activity levels compared with younger individuals (median 3 vs. 2 months, *p* < 0.001), despite comparable pain scores throughout follow-up. This delay is consistent with prior observations emphasizing the impact of pre-injury physical conditioning and physiological resilience on postoperative recovery [16). Multivariate analysis identified delayed surgery (OR 1.341, 95% CI 1.123–1.602, *p* = 0.001), prolonged hospitalization (OR 1.059, 95% CI 1.002–1.119, *p* = 0.044), and postoperative complications (OR 1.941, 95% CI 1.043–3.610, *p* = 0.036) as independent predictors of chronic pain, while age itself showed no association. These findings indicate that modifiable perioperative factors—rather than chronological age—drive long-term pain outcomes, and underscore the importance of timely surgical intervention, structured rehabilitation, and complication prevention in optimizing recovery for elderly SSRF patients [6, 10, 11].

Although age did not emerge as an independent predictor of adverse outcomes in multivariable analysis, this finding must be interpreted within the context of selective surgical candidacy. Patients with severe frailty, multiorgan failure, or limited physiological reserve were likely excluded from SSRF consideration, constraining the generalizability of age-neutral outcomes. The absence of standardized frailty assessment represents a key limitation of this study, as baseline functional reserve—rather than chronological age—may be a more meaningful determinant of postoperative risk. Discrepancies in the literature, such as the markedly elevated mortality reported in studies where frailty was not systematically evaluated, further highlight the necessity of incorporating validated frailty indices into preoperative risk stratification. Future prospective studies integrating formal frailty assessment are essential to refine patient selection algorithms and optimize outcomes in elderly SSRF candidates [8].

Additional limitations include the retrospective design and reliance on self-reported pain and functional outcomes, which may not fully capture long-term quality-of-life impacts. **Functional recovery was assessed through structured patient interviews rather than validated functional or quality-of-life instruments**,** which represents an inherent limitation; therefore**,** the observed delay in functional recovery in elderly patients should be interpreted as a descriptive finding that may reflect expected age-related recovery patterns rather than a clearly defined clinically meaningful impairment.** Low mortality rates reduced statistical power for modeling rare events. Moreover, this cohort represents surgically selected elderly trauma patients; frail individuals with limited physiological reserve or early trauma-related mortality were not candidates for SSRF and are not represented, limiting generalizability to the broader geriatric trauma population. The study was conducted in line with STROBE guidelines.

## Conclusion

SSRF demonstrated comparable safety and clinical outcomes in geriatric and non-geriatric patients, suggesting that advanced age alone should not necessarily preclude surgical stabilization in appropriately selected individuals. Chronic pain was mainly associated with modifiable perioperative factors—delayed surgery, prolonged hospitalization, and complications—rather than age itself, while functional recovery was slower in elderly patients despite similar pain trajectories and complication rates. However, this study included only surgically selected patients without a non-operative control group; therefore, no conclusions can be drawn regarding comparative effectiveness versus conservative management, and findings apply only to carefully selected surgical candidates

## Supplementary Information

Below is the link to the electronic supplementary material.

Supplementary Material 1

## References

[1] Bulger EM, Arneson MA, Mock CN, Jurkovich GJ (2000) Rib fractures in the elderly. J Trauma - Injury Infect Crit Care 48(6):1040–1046 10.1097/00005373-200006000-00007 10866248

[2] Qureshi I, Kharel R, Mujahid N, Neupane I (2023) Rib Fracture Management in Older Adults: A Scoping Review. J Brown Hosp Med. ;2(3) 10.56305/001c.82211 PMC11864390 40026458

[3] Mai HT, Tran TS, Ho-Le TP, Pham TT, Center JR, Eisman JA et al (2018) Low-trauma rib fracture in the elderly: Risk factors and mortality consequence. Bone 116:295–300 30172740 10.1016/j.bone.2018.08.016

[4] Coary R, Skerritt C, Carey A, Rudd S, Shipway D (2020) New horizons in rib fracture management in the older adult. Age Ageing 49(2):161–167 31858117 10.1093/ageing/afz157

[5] Sawyer E, Wullschleger M, Muller N, Muller M (2022) Surgical Rib Fixation of Multiple Rib Fractures and Flail Chest: A Systematic Review and Meta-analysis. J Surg Res 276:221–234 35390577 10.1016/j.jss.2022.02.055

[6] Sermonesi G, Bertelli R, Pieracci FM, Balogh ZJ, Coimbra R, Galante JM et al (2024) Surgical stabilization of rib fractures (SSRF): the WSES and CWIS position paper. World J Emerg Surg 2024 19(1):1–35 10.1186/s13017-024-00559-2 PMC11487890 39425134

[7] Meyer DE, Harvin JA, Vincent L, Motley K, Wandling MW, Puzio TJ et al (2023) Randomized Controlled Trial of Surgical Rib Fixation to Nonoperative Management in Severe Chest Wall Injury. Ann Surg 278(3):357–365 37317861 10.1097/SLA.0000000000005950 PMC10527348

[8] Duong W, Grigorian A, Nahmias J, Farzaneh C, Christian A, Dolich M et al (2022) An increasing trend in geriatric trauma patients undergoing surgical stabilization of rib fractures. Eur J Trauma Emerg Surg 48(1):205–210 33095279 10.1007/s00068-020-01526-7 PMC7583690

[9] Chen Zhu R, De Roulet A, Ogami T, Khariton K (2020) Rib fixation in geriatric trauma: Mortality benefits for the most vulnerable patients. J Trauma Acute Care Surg 89(1):103–110 32176172 10.1097/TA.0000000000002666

[10] Varre JSV, Hopmann P, Wu JL, Bach JA, Suh KI, Goslin BJ et al (2023) In- and out-of-hospital outcomes following surgical stabilization of rib fractures in 80 years and older: A single-institution experience. Injury. ;54(9) 10.1016/j.injury.2023.110871 37353448

[11] Jensen S, Sanderfer VC, Porter K, Rieker MG, Maniscalco BR, Lloyd J et al (2024) Surgical stabilization of rib fractures in the geriatric trauma population is associated with equivalent outcomes to a younger cohort: A propensity matched analysis. Injury. ;55(7) 10.1016/j.injury.2024.111593 38762943

[12] Tyburski JG, Collinge JD, Wilson RF, Eachempati SR (1999) Pulmonary contusions: quantifying the lesions on chest X-ray films and the factors affecting prognosis. J Trauma 46(5):833–838 10338400 10.1097/00005373-199905000-00011

[13] Chapman BC, Herbert B, Rodil M, Salotto J, Stovall RT, Biffl W et al (2016) RibScore: A novel radiographic score based on fracture pattern that predicts pneumonia, respiratory failure, and tracheostomy. J Trauma Acute Care Surg 80(1):95–101 26683395 10.1097/TA.0000000000000867

[14] Rating the Severity of Tissue Damage (1971) I. The Abbreviated Scale. JAMA: J Am Med Association 215(2):277–280 10.1001/jama.1971.03180150059012 5107365

[15] Mörs K, Wagner N, Sturm R, Störmann P, Vollrath JT, Marzi I et al (2021) Enhanced pro-inflammatory response and higher mortality rates in geriatric trauma patients. Eur J Trauma Emerg Surg 47(4):1065–1072 31875239 10.1007/s00068-019-01284-1

[16] Choi J, Marafino BJ, Vendrow EB, Tennakoon L, Baiocchi M, Spain DA et al (2021) Rib Fracture Frailty Index: A risk stratification tool for geriatric patients with multiple rib fractures. J Trauma Acute Care Surg 91(6):932–939 34446653 10.1097/TA.0000000000003390

