# Supplementary material for: Surgical stabilization of rib fractures in older adults: a retrospective cohort study
Source: Updates Surg. 2026 Mar 19;78(4):1757–63. doi: 10.1007/s13304-026-02597-8 (PMC13421296; doi:10.1007/s13304-026-02597-8)
Supplement: Supplementary file 1 — Supplementary Material 1 [file 13304_2026_2597_MOESM1_ESM.docx]

| **Characteristic** | **Non-Geriatric (<70 years) n=397** | **Geriatric (≥70 years) n=127** | **p-value** |
| --- | --- | --- | --- |
| Age, mean (SD) | 54.16 (10.43) | 75.96 (5.31) | <0.001 |
| Male sex, n (%) | 329 (82.9) | 104 (81.9) | 0.799 |
| BMI, mean (SD) | 26.29 (4.1) | 26.17 (3.9) | 0.951 |
| **Injury Mechanism, n (%)** |  |  |  |
| Fall | 190 (47.9) | 87 (68.5) | <0.001 |
| Traffic accident | 143 (36) | 33 (26) | 0.034 |
| **Injury Characteristics** |  |  |  |
| Number of ribs fractured, median (IQR) | 4 (1-11) | 4 (1-8) | 0.203 |
| Flail chest, n (%) | 65 (16.4) | 17 (13.4) | 0.414 |
| Bilateral fractures, n (%) | 64 (16.2) | 22 (17.3) | 0.867 |
| First rib fracture, n (%) | 51 (12.9) | 11 (8.7) | 0.201 |
| **Intrathoracic Complications** |  |  |  |
| Pulmonary contusion, n (%) | 250 (63.1) | 95 (74.8) | 0.013 |
| Pneumothorax, n (%) | 117 (29.5) | 35 (27.6) | 0.668 |
| Hemothorax, n (%) | 207 (52.3) | 78 (61.4) | 0.01 |
| **Injury Severity Scores** |  |  |  |
| AIS-Thorax, mean (SD) | 2.94 (0.748) | 3.0 (0.735) | 0.348 |
| RibScore, mean (SD) | 1.94 (1.158) | 1.91 (1.54) | 0.789 |
| BPC18, mean (SD) | 1.36 (1.22) | 1.52 (1.09) | 0.122 |

Table 1. Demographic Characteristics, Injury Mechanisms, and Injury Severity Profiles of the Study Cohort

Table 2: Comparison of Operative Characteristics and Postoperative Outcomes Between Geriatric and Non-Geriatric Patients

| **Outcome** | **Non-Geriatric n=397** | **Geriatric n=127** | **p-value** |
| --- | --- | --- | --- |
| **Operative Characteristics** |  |  |  |
| Time to surgery, median days (IQR) | 2 (1-10) | 2 (1-8) | 0.324 |
| Number of ribs operated, mean (SD) | 4.19 (1.59) | 4.53 (1.58) | 0.02 |
| Number of plates used, median (IQR) | 4 (1.71) | 4 (2.02) | 0.026 |
| Bilateral surgery, n (%) | 11 (2.8) | 1 (0.8) | 0.192 |
| **Postoperative Outcomes** |  |  |  |
| Overall complications, n (%) | 53 (13.6) | 16 (12.7) | 0.798 |
| - Pneumonia | 19 (4.8) | 8 (6.3) | 0.502 |
| - Hardware complications | 17 (4.3) | 5 (3.9) | 0.55 |
| - Surgical site infection | 7 (1.8) | 1 (0.8) | 0.97 |
| ICU admission, n (%) | 107 (27) | 35 (27.6) | 0.905 |
| ICU stay, mean days (SD) | 1.58 (4.28) | 1.12 (3.4) | 0.797 |
| Hospital stay, mean days (SD) | 8.74 (4.42) | 8.66 (4.13) | 0.996 |
| 30-day readmission, n (%) | 18 (4.5) | 6 (4.7) | 0.933 |

Table 3: Comparison of Postoperative Pain Scores and Functional Recovery Between Geriatric and Non-Geriatric Patients

| **Outcome** | **Non-Geriatric n=397** | **Geriatric n=127** | **p-value** |
| --- | --- | --- | --- |
| **Pain Scores (0–10 scale)** |  |  |  |
| 1-month, mean (median) | 2.61 (3) | 2.61 (3) | 0.944 |
| 3-month, mean (median) | 1.37 (1) | 1.36 (1) | 0.989 |
| 6-month, mean (median) | 0.62 (0) | 0.58 (0) | 0.901 |
| **Functional Outcomes** |  |  |  |
| Chronic pain (>6 months), n (%) | 64 (16.7) | 26 (21.5) | 0.227 |
| Return to activity, median months | 2 | 3 | 0.02 |

Table4: Correlation Analysis Between Injury Severity Indices (BPC18, RibScore, AIS-Thorax) and Key Clinical Outcomes

| **Clinical Outcome** | **BPC18 r / p-value** | **RibScore r / p-value** | **AIS-Thorax r / p-value** |
| --- | --- | --- | --- |
| **Hospital Outcomes** |  |  |  |
| ICU length of stay | 0.375 / <0.001 | 0.412 / <0.001 | 0.350 / <0.001 |
| Total hospital stay | 0.191 / <0.001 | 0.206 / <0.001 | 0.232 / <0.001 |
| Return to normal activity | 0.036 / 0.488 | 0.185 / <0.001 | 0.104 / 0.042 |
| **Pain Outcomes** |  |  |  |
| 1st month pain score | 0.064 / 0.211 | 0.137 / 0.007 | 0.094 / 0.067 |
| 3rd month pain score | 0.050 / 0.331 | 0.164 / 0.001 | 0.124 / 0.016 |
| 6th month pain score | 0.037 / 0.465 | 0.97 / 0.058 | 0.102 / 0.046 |
| **Binary Outcomes (p-value)** |  |  |  |
| Chronic pain presence | 0.382 | 0.027 | 0.025 |
| Postoperative complications | 0.045 | 0.027 | 0.346 |
| In-hospital mortality | 0.252 | 0.220 | 0.222 |

|  | | | | |
| --- | --- | --- | --- | --- |
| Variable | Univariate OR (95% CI) | p-value | Multivariate OR (95% CI) | p-value |
| **Chronic Pain Predictors** |  |  |  |  |
| Day of surgery after admission | 1.425 (1.202–1.690) | <0.001 | 1.341 (1.123–1.602) | 0.001 |
| No. of fractured ribs (operated side) | 1.154 (1.020–1307) | 0.023 | 1.059 (1.002–1.119) | 0.044 |
| Total hospital length of stay | 1.091 (1.038-1.146) | <0.001 | 1.941 (1.043–3.610) | 0.036 |
| Postoperative complications | 2.316 (1.304–4.114) | 0.004 | 1.025 (0.839–1.252) | 0.169 |
| AIS-Thorax | 1.418 (1.004–2.003) | 0.047 | 1.160 (0.666–2.021) | 0.599 |
| **Complication Predictors** |  |  |  |  |
| No. of fractured ribs (operated side) | 1.339 (1.171–1.531) | 0.104 | 2.014(1.493-2717) | <0.001 |
| Preoperative ventilator use | 1.241 (0.923–1.668) | 0.153 | NS | NS |
| Total number of plates | 1.133 (0.998–1.285) | 0.053 | NS | NS |
| RibScore | 1.147 (0.991–1.327) | 0.065 | NS | NS |
| BPC18 | 1.209 (0.986–1.483) | 0.068 | NS | NS |
| First rib fracture | 2.358 (1.235–4.504) | 0.009 | 3.303 (1.025-10.646) | 0.045 |
| Male sex | 0.544 (0.300–0.985) | 0.044 | NS | NS |

Table 5: Predictors of Chronic Pain and Postoperative Complications: Univariate and Multivariate Logistic Regression Analysis
